# Effect of Feeding Lactating Ewes with *Moringa oleifera* Leaf Extract on Milk Yield, Milk Composition and Preweaning Performance of Ewe/Lamb Pair

**DOI:** 10.3390/ani10071117

**Published:** 2020-06-29

**Authors:** Gabriel Olvera-Aguirre, Miriam Marleny Mendoza-Taco, Darwin Nicolas Arcos-Álvarez, Angel Trinidad Piñeiro-Vázquez, Victor Manuel Moo-Huchin, Jorge Rodolfo Canul-Solís, Luis Castillo-Sánchez, Marco Antonio Ramírez-Bautista, Einar Vargas-Bello-Pérez, Alfonso Juventino Chay-Canul

**Affiliations:** 1 División Académica de Ciencias Agropecuarias, Universidad Juárez Autónoma de Tabasco, km 25. Carretera Villahermosa-Teapa, R/A La Huasteca., Colonia Centro Tabasco 86280, Mexico; 13030027@itesa.edu.mx (G.O.-A.); marmirlen@hotmail.com (M.M.M.-T.); 2 Tecnológico Nacional de México, Instituto Tecnológico de Conkal, Conkal, Yucatán 97345, Mexico; darwin.arcos@itconkal.edu.mx (D.N.A.-Á.); angel.pineiro@itconkal.edu.mx (A.T.P.-V.); 3 Tecnológico Nacional de México, Instituto Tecnológico de Mérida, km 5 Mérida-Progreso, Mérida, Yucatán 97120, Mexico; vmmoo@yahoo.com; 4 Tecnológico Nacional de México, Instituto Tecnológico de Tizimín, Tizimín, Yucatán 97000, Mexico; jcanul31@gmail.com (J.R.C.-S.); luis.castillo@ittizimin.edu.mx (L.C.-S.); 5 Tecnológico Nacional de México, Instituto Tecnológico de Chiná, Chiná, Campeche 24520, Mexico; tonorb75@hotmail.com; 6 Department of Veterinary and Animal Sciences, Faculty of Health and Medical Sciences, University of Copenhagen, Grønnegårdsvej 3, DK-1870 Frederiksberg C, Denmark

**Keywords:** performance, lactation, herbal extract, milk composition, milk yield, performance, supplementation in lactating ewes

## Abstract

**Simple Summary:**

The use of plant extracts as supplemental additives in ruminant diets shows beneficial effects. This study evaluated the effects of different doses of *Moringa oleifera* leaf extract (MOE) on milk production and milk composition in ewes and on preweaning performance of their lambs. At different doses, MOE supplementation did not affect overall productive traits in ewes and lambs and did not have negative effects on milk production and milk quality.

**Abstract:**

The objective this study was to evaluate the effect of different doses of *Moringa oleifera* leaf extract (MOE) on milk production and milk composition in ewes and on preweaning performance of their lambs. Twenty-four lactating ewes were housed individually with their lambs and assigned to four groups in a completely randomized design. The treatments included a basal diet without MOE (MOE0) or a basal diet supplemented with either 20 mL MOE per ewe per day (MOE20), 40 mL MOE per ewe per day (MOE40) or 60 mL MOE per ewe per day (MOE60). Over 45 days, milk production was recorded weekly and individual milk samples were collected for chemical analysis. Milk yield, fat-corrected milk and daily yields were similar among the four treatments. The supply of MOE did not affect ewe weaning efficiency and average daily gain or litter weaning weight of the lambs. Overall, the results from this study showed that dietary supplementation of hydroalcoholic extracts of *Moringa oleifera* leaves at doses of 20, 40 or 60 mL/ewes/d in lactating ewes does not have negative effects on milk yield, milk composition or lamb performance.

## 1. Introduction

In recent years, the use of agricultural byproducts, herbal plants and/or their extracts as feed additives in ruminant production systems has increased due to their relative low cost, their potential to replace synthetic products and consumers’ high demand for organic animal products [1,2,3,4]. Agroindustrial byproducts consist of considerable amounts of bioactive components, such as polyphenols (tannins or flavonoids), and these compounds exert antimicrobial activities to improve immune status and reduce stress [3,4]. Medicinal plants are used in animal nutrition to improve performance, feed efficiency, nutrient utilization, overall animal health and quality of livestock products, which are effects related to their abilities to increase oxidative stability [4,5,6].

During lactation, adequate energy levels in the diets of ewes improves body condition score, body weight, milk yields and, consequently, weight gain of their lambs [6,7]. In this context, some authors reported that supplementation of lactating ewes’ diets with foliage and meal obtained from leaf and other plant components improved milk yield, milk fat and milk fatty acid profiles [8,9]. Some authors reported that due to their oxidative stability, phenolic compounds, tannins, saponins and flavonoids could be used to improve the functional properties of milk, meat and derived products of small ruminants [4,8,10,11].

Recently, interest focused on *Moringa oleifera* (MO), a highly versatile and sustainable tree mostly cultivated in the tropics and subtropics [12]. MO is very useful as a feed supplement for animals [10,13,14] and is an important source of protein (up to 35% of dry matter, DM) and fibre (up to 28% DM) [15,16,17]. In addition to its nutritional properties, MO leaves are sources of bioactive compounds, such as antioxidants, vitamins, phenolic acids and flavonoids, and are available at relatively low cost in resource-limited areas, making it a promising source of bioactive compounds for functional food production and nutraceutical product development [12,14].

Several authors [1,16,18,19] reported that dietary supplementation with MO leaves or extracts increased dry matter intake (DMI), total solids in milk, milk nonfat solids, milk fat, daily milk yield (DMY) and energy-corrected milk yield (ECM) in goats. Moreover, Moyo et al. [20] concluded that supplementation with MO leaves in goat diets protected animals against diseases induced by oxidative stress, as shown in liver enzymes. Until now, no study determined whether supplementing ewes with MO extracts impacted both the dam and the lamb preweaning performances. We hypothesized that supplementation of lactating ewe diets with MOE would be a useful strategy to improve ewe performance and milk composition, with positive consequences on suckling lamb performance. Therefore, the objective of this study was to evaluate the effects of supplementing lactating ewes with increasing doses of MO leaf extracts on milk yield and milk composition, and on the preweaning performance of ewe/lamb pair. The choice to use extracts rather than plant parts from MO was due to the extracts being concentrated solutions of secondary plant compounds, and their delivery and supply is constant all year round.

## 2. Materials and Methods

### 2.1. Management of Animals and Experimental Design

The animals were treated in accordance with the guidelines and regulations for animal experimentation of the División Académica de Ciencias Agropecuarias, Universidad Juárez Autónoma de Tabasco (ID project PFI: UJAT-DACA-2015-IA-02). The study was carried out at the “Rancho San Francisco”, located at 21°14′48″ N and 89°02′35″ W longitude, 5 m above sea level (masl), in Dzidzantun municipality (Yucatán, Mexico). The average temperature is 26 °C, with 9.8 mm of rainfall during the experimental months (between November and December) and extremes of relative humidity between 66% and 89% [21,22]. Twenty-four clinically healthy crossbreed (Pelibuey × Katahdin) ewes that had recently lambed (2 days) were randomly assigned to four groups of six animals each. Ewes were 2–3 years old with a mean body weight (BW) of 35.7 ± 5.02 kg and a body condition score (BCS) of 2.07 ± 0.18 [23] with a single lambing. The number of animals for each treatment was based on a similar study dealing with dietary interventions [24].

After lambing, each ewe and their offspring were allocated together in individual pens (2 × 3 m) and managed under a feedlot system for 45 days. The ewes had free access to feed and water. For the lambs, water was always available, but they did not have access to the ewes’ feeders. At the beginning of the study, ewes were dewormed with Closantel 5%^®^ (Wyeth LLC, Madison, NJ, USA) at a dosage of 10 mg/kg of body weight.

The basal diet consisted of a concentrate (offered at 8:00 am) based on ground corn, soybean meal, sugarcane molasses and minerals and fresh chopped Taiwan grass (*P. purpureum*; offered at 18:00 h), using only stems in order to reduce the nutritional variation throughout the experimental phase, in a proportion of 80:20 (concentrate:forage) ratio. The basal diet had an estimated metabolizable energy of 11.5 MJ/kg DM and 15% crude protein [25] (Table 1) to meet the energy requirements and to avoid ewe weight loss, thereby maintaining the body condition score. This strategy was performed to prevent body fat mobilization and possible confounding effects on milk composition. The objective was to maintain constant BW and BCS of ewes throughout the study, as described previously [26]. Throughout the experiment, the diet was offered ad libitum, with feeding levels designed to ensure a daily refusal margin of 10%. The dry matter intake (DMI) was determined by the difference between the amount of diet offered and the amount refused. Animals had free access to water. The basal diet was supplemented with *Moringa oleifera* leaves extract (MOE) provided at doses (per ewe daily) of either 0 mL (control), 20 mL (ME20), 40 mL (ME40) or 60 mL (ME60). The MOE was supplied daily in the concentrate. In order to avoid feed sorting, treatments were manually mixed with 200 g of concentrate and, after animals consumed it, the rest of the meal was provided. The diets were formulated to meet the theoretical requirements for dairy ewes with a mean BW of 45 kg and a mean milk yield of 1.74 kg/d, with milk total protein and fat contents of 4.5% and 7.0%, respectively, according to the Agricultural and Food Research Council [25] guidelines.

The BW of each ewe was recorded weekly. The amount of feed offered to each ewe was adjusted weekly according to BW [23]. The data collected also included initial (IBW, kg) and final (FBW, kg) body weights from each ewe. Calculated traits included daily preweaning weight gain of lambs (ADG, kg), average daily body weight change of ewes (BWC, kg) during lactation, which was estimated as the difference between FBW and IBW divided by 45 days, and litter weaning weight (LWW, kg), which was was obtained as the sum of weight of weaned lambs by ewe. Ewe weaning efficiency (EE) was calculated as LWW/FBW × 100 [23].

### 2.2. Milk Yield and Composition

Milk yield (MY, kg) and lamb body weights (LBW, kg) were measured every week (on Saturdays) from the first week of the experiment until day 45 (6 weeks). The lambs were separated from their dam at 19:00 h. After 12 h of separation, the ewes were hand-milked after an intramuscular injection of 3 international units of oxytocin (Pisa, Mexico). Before milking, the teats were cleaned with an iodine solution and dried with paper towels. Daily milk yield (DMY, kg) of ewes during the week of measurement was calculated by the milk obtained over the 12 h period, which was multiplied by 2 to reflect the DMY [26].

For milk composition analysis, samples from each ewe (100 mL) were obtained every week. Analyses for fat, protein and lactose were performed in duplicate using an automatic milk analyzer (Lactoscan LS-60, Milkotronic Ltd., Nova Zagora, Bulgaria). The equipment was calibrated for fat by the Gerber method and for protein by total nitrogen determination according to the Dumas method using a LECO CNS-2000 series 3740 analyzer (LECO Corp. St. Joseph, MI, USA). Additionally, fat-corrected milk yield (FCM) to 6% was calculated according to the formula [27]:

(1) FCMY = (0.28 + 0.12F) × MY

where F = fat percentage. Energy-corrected milk yield (ECMY, kg) was calculated according to the formula [28]

(2) ECMY = (0.071 × F + 0.043 × P + 0.2224) × MY

where P is protein percentage.

### 2.3. Moringa oleifera Leaf Extract

The MOE was prepared at the beginning of the experiment and stored at 20 °C in a dark room (to avoid oxidation) for daily use. The MO leaves were randomly collected from young and mature plants. Leaves were cut (1 to 2 cm long) and dried at 40 ° C for 72 h in a forced convection oven. The dried leaves were ground with a mill (0.5–1 mm) and the resulting powder was immediately processed. The leaf powder was subjected to extraction with a 50% aqueous ethanol in a ratio of 1:20 for 2 h under continuous stirring (120 rpm) at 25 ° C. The resulting extract was centrifuged at 1500 rpm for 10 min at 25 °C. The supernatant was collected and the pellet was subjected to additional extraction using the same procedure as described above. Supernatants from both extractions were pooled and finally stored.

### 2.4. Antioxidant Content

The contents of MOE total saponins, hydrolyzable tannins, condensed tannins, total phenolic compounds and total flavonoids were determined by spectrophotometry. Furthermore, in vitro antioxidant activity was determined by 2,2-diphenyl-1-picrylhydrazyl (DPPH) and 2,2′-azinobis(3-ethylbenzothiazoline-6-sulfonic acid) (ABTS) assays. In both methods, the DPPH and ABTS radicals were reduced by hydrogen donation of the antioxidant.

### 2.5. Total Saponins

Total saponins content was determined as described by Ncube et al. [29]. Samples containing 250 µL of extract and 250 µL of vanillin reagent (8%, in ethanol) were mixed and 2.5 mL of sulfuric acid was added (72%, *v*/*v*). The solution was mixed in a vortex and placed in a water bath at 60 °C for 10 min. The test tubes were allowed to cool down for 4 min and absorbance was measured at 544 nm using a UV-Vis spectrophotometer Agilent Technologies Cary 60 (Santa Clara, CA, USA). Total saponins were expressed as milligrams of diosgenin equivalents per 100 mL of plant extract (mg DE/100 mL). The calibration curve of diosgenin showed a linearity range from 80 to 800 ppm (r^2^ > 0.99).

### 2.6. Condensed Tannins

Condensed tannins were determined based on the vanillin–HCl method described by Selcuk and Erkan [30]. A 0.5 mL extract sample was mixed with 3 mL of vanillin reagent (4%, *w*/*v*, in methanol) and 1.5 mL of concentrated HCl was added. The mixture were stirred in a vortex, the solution was kept in the dark for 15 min at 25 °C and the absorbance was read at 500 nm using a UV-Vis spectrophotometer Agilent Technologies Cary 60 (Santa Clara, CA, USA). Total condensed tannins were expressed as milligrams of catechin equivalents per 100 mL of plant extract (mg CE/100 mL). The calibration curve of catechin showed a linearity range from 5 to 300 ppm (r^2^ > 0.99).

### 2.7. Hydrolysable Tannins

Hydrolyzable tannins were determined based on the method described by Çam and Hışıl [31]. A 1 mL extract sample was mixed with 5 mL of KIO_3_ (2.5%, *w*/*v*, in water) (previously exposed for 7 min at 30 °C). The mixture was exposed to 30 °C for 2 min and the absorbance was measured at 550 nm using a UV-Vis spectrophotometer Agilent Technologies Cary 60 (Santa Clara, CA, USA). Hydrolyzable tannins were expressed as milligrams of tannic acid equivalents per 100 mL of plant extract (mg TAE/100 mL). The calibration curve of tannic acid showed a linearity range from 500 to 5000 ppm (r^2^ > 0.99).

### 2.8. Total Phenolic Compounds

The amount of total phenolic compounds was determined based on the methods described by Moo-Huchin et al. [32]. A total of 50 µL of extract was mixed and equilibrated for 5 min with 3 mL of distilled H_2_O and 250 µL of Folin–Ciocalteu reagent (1 N). After equilibrium, 750 µL of NaCO_3_ (20%, *v*/*v*, H_2_O) was added, followed by 950 µL of distilled H_2_O. The solution was stirred and incubated for 30 min at 25 °C and the absorbance was read at 765 nm using a UV-Vis spectrophotometer Agilent Technologies Cary 60 (Santa Clara, CA, USA). Total phenolic compounds were expressed as milligrams of gallic acid equivalents per 100 mL of plant extract (mg GAE/100 mL). The calibration curve of gallic acid showed a linearity range from 100 to 1000 ppm (r^2^ > 0.99).

### 2.9. Total Flavonoid Contents

Flavonoid contents were determined based on the methods described by Moo-Huchin et al. [32], where 1 mL of extract was mixed and equilibrated with 4 mL of distilled H_2_O and 300 µL 5% NaNO_2_ for 5 min. After equilibrium, 300 µL of 10% AlCl_3_ (methanol solution) was added. The mixture was kept for 1 min and then 2 mL of 1 M NaOH was added. The last volume was made up to 10 mL with distilled H_2_O. The mixture absorbance was determined at 415 nm using a UV-Vis spectrophotometer Agilent Technologies Cary 60 (Santa Clara, CA, USA). The concentration of total flavonoids was calculated using a standard curve of quercetin 0–1000 ppm (r^2^ > 0.99) and expressed as mg of quercetin equivalents (QE)/100 mL of plant extract.

### 2.10. Antioxidant Activity

The DPPH (2,2-diphenyl-1-picrylhydrazyl) assay was conducted according to Moo-Huchin et al. [32], where 100 µL extract was added to 3.9 mL of freshly prepared DPPH solution (6 × 10^−5^ M in methanol). The mixtures were shaken and kept in the dark at 25 °C for 30 min. The mixture absorbance was determined at 515 nm. A calibration curve was prepared using Trolox as the standard and the results were expressed as mM Trolox equivalents/100 mL of extract.

The ABTS (2,2-Azinobis-3-ethylbenzotiazoline-6-sulphonic acid) assay was conducted according to Moo-Huchin et al. [32]. The ABTS radical was formed via the reaction between 7 mM ABTS^+^ solution and 140 mM potassium persulfate solution. The solutions were incubated in the dark at 25 °C for 18 h. The ABTS-activated radical was diluted with ethanol to an absorbance of 0.70 ± 0.02 at 734 nm. Then, 30 µL extract was transferred to a test tube with 2970 µL of the ABTS radical. The absorbance was determined at 734 nm after 30 min. Trolox was used as a standard and the results were expressed as mM Trolox equivalents/100 mL of extract.

### 2.11. Statistical Analyses

Data on ewe performance traits (total milk yield TMY, DMY, BWC, LWW and EE) and lamb performance traits (BW and ADG) were analyzed using a completely randomized design by analysis of variance considering treatments as fixed effects and the ewe or lamb as random effects. Tukey’s test was performed when a significant treatment effect (*p* < 0.05) was detected. Linear (*L*) and quadratic (*Q*) effects of treatments were tested for the response variables. All statistical analyses were performed using the Statistical Analysis System software [33].

Repeated measures of ewe milk production and milk components were analyzed using the MIXED procedure with a mixed linear model that included the fixed effect of sampling day, treatment, two-way interaction of treatment × sampling day and the random effect of ewe. Repeated measures using the same animal were modeled assuming a compound symmetry (CS) covariance structure.

The linear expression of the model was

$$Y_{ijklm}=\mu +Ewe_{i}+Trat_{j}+Dm_{k}+Trat*Dm_{l}+\varepsilon_{ijklm}$$

where $Y_{ijklm}$ represents TMY, DMY, ECM…, μ is the overall mean, $Ewe_{i}$ is the random effect of the *i-*th ewe, $Trat_{j}$ is the fixed effect of the *j-*th treatment, $Dm_{k}$ is the fixed effect of the *k-*th Day of measurement, $Treat*Dm_{l}$ is the fixed effect of the *l-*th treatment × day measurement interaction and $\varepsilon_{ijklm}$ is the residual random effect.

## 3. Results

At the end of the experiment (last three weeks), one animal from ME40 treatment was removed from the experiment due to illness associated with locomotion problems. These data were not included in the analysis.

### 3.1. Moringa oleifera Extracts

The yield obtained by successive hydroalcoholic extractions under orbital agitation was 1.34 ± 0.009%. The MOE phytochemical assays showed high concentrations of bioactive compounds (Table 2) in the form of antioxidants (tannins, phenols and flavonoids), with a total phenolic content of 140.41 mg of galic acid equivalent (GAE). This resulted in a great capacity to capture free radicals, as demonstrated by the amount of Trolox eq 100 mL^-1^ of the extract supported by the DPPH and ABTS tests (Table 2).

### 3.2. Ewe and Lamb Performance

**Figure 1:**
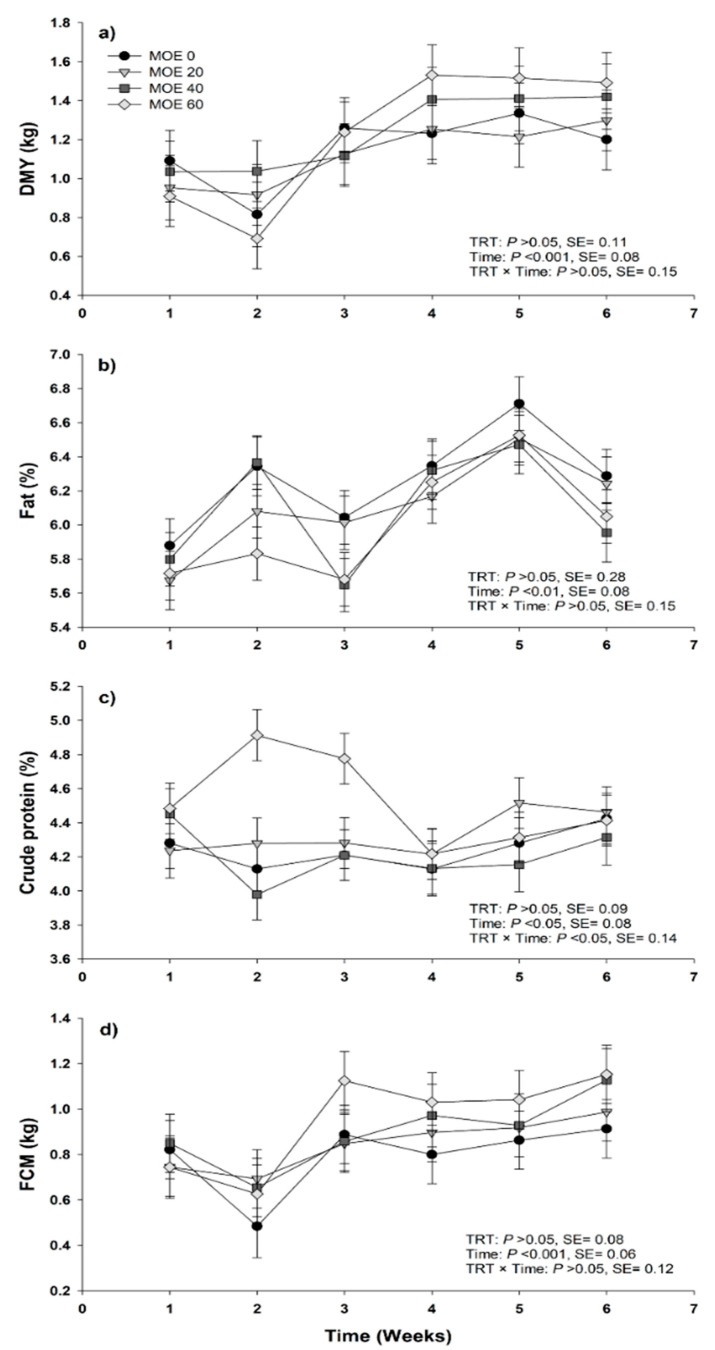
Daily milk yield (DMY) (**a**), milk fat (**b**), milk protein (**c**) and fat-corrected milk (FCM) at 6% (**d**) from animals supplemented with *Moringa oleifera* leaves extract provided at doses (per ewe daily) of either 0 mL (control), 20 mL (ME20), 40 mL (ME40) or 60 mL (ME60). Bars denote standard error of the means.

No significant effects between treatments were observed regarding TMY, DMY, milk components or daily yield (Table 3). Daily milk yield, fat-corrected milk 6% (FCM6), milk fat and milk protein varied over time (Figure 1). The DMI (kg d^-1^) was higher (*p* = 0.02) for MOE40 compared with MOE60, however, this did not differ from MOE0 and MOE20. Also, DMI (g kg ^0.75–1^ d^−1^) was greater (*p* < 0.001) for MOE40 compared with MOE20 and MOE60, but did not differ from MOE0 (Table 4). In addition, BWC, LWW and EE were similar among treatments (*p* > 0.05). Likewise, there was no difference in ADG (*p* > 0.05) or weaning weight of lambs at 45 days between treatments (Table 4).

## 4. Discussion

### 4.1. Moringa oleifera Extracts

In this study, the obtained yield for MO hydroalcoholic extract was 1.34%, which was lower than yields reported by Vongsak et al. (7.9%) [34] and Saleem et al. (40.5%) [35] in aqueous solutions with methanol and hydroalcoholic extracts (70% ethanol–water). However, the contents of bioactive compounds and antioxidant activity found in the present study were comparable to previous work [20,35,36] using aqueous solutions with methanol and acetone MO leaf (fresh and dry) extracts. The most important components of MO leaf extracts are phenols, tannins and flavonoids, such as quercetin, catechin, tannic acid and gallic acid, which exhibit biological and pharmaceutical activities (e.g., antibiotic, anti-inflammatory, antioxidative). Some authors [20] reported that the MO acetone and water extracts presented inhibition percentages in ABTS and DPPH of 95.27% and 72.89% and 98.24% and 83.56%, respectively, compared to butylhydroxytoluene (98% inhibition) at 1 mg mL^−1^, with total phenols of 120.33 and 40.27 and total flavonoids of 295.01 and 45.1 mg g^−1^. These high antioxidant activity levels attributed to polyphenolic compounds suggest that they could be used as supplements to prevent oxidative stress-related diseases in animals.

### 4.2. Milk Production

In underfed ruminants or during periods of high nutrient demand, such as gestation and lactation periods, if nutritional requirements are not fulfilled, oxidative stress is generated, consequently producing disease states, low production performance, higher mortality rates and poor product quality [37]. With regard to production performance, some authors [13,18,19] reported an increase in milk production, total solids in milk and nonfatty solids when supplementing lactating ruminants with MO as a strategy for supplying dietary antioxidants. Also, when supplying MO leaf meals to lactating goats [18], increases in DMI, MY, energy-corrected milk, fat, protein and lactose were observed. However, when replacing alfalfa with MO leaves in lactating Nadji ewes, [13] DMY was not affected during the adaptation period, but it did increase between the third and sixth weeks of lactation. In the Nadji ewes study, no significant differences between treatments were observed in milk composition, but numerical increases in milk fat (17%), lactose (16%) and energy output (35%) were observed. The authors concluded that dietary inclusion of MO in Najdi ewes improved milk yield and milk quality. On the other hand, [14] supplemented Jersey cows with 30 and 60 g of MO meal per animal per day exhibited no differences in DMY, protein or lactose, but fat increased at a dose of 60 g d^−1^ compared to the control group.

In the present study, MO hydroalcoholic extracts were used as supplements in lactating sheep. Although the use of alcohol as a solvent may cause detrimental effects in animals, several authors pointed out that alcohol consumption in sheep is common when providing fermented feeds, which reach concentrations of 10 to 50 g kg^-1^ [38,39]. They also pointed out that in ruminants, alcohol is naturally metabolized by fungi and bacteria in the rumen, and these animals can easily metabolize 150 to 180 mg/kg BW due to the presence of alcohol dehydrogenase in their body [38]. Alcohol doses between 0.2 to 1 g BW EtOH d^-1^ were used in sheep [39]. When adding 5% ethanol or acetic acid to the diets of dairy cows, Daniel et al. [39] found differences in DMI, milk fat and milk protein, however, DMY showed an improved response when supplemented with 5% ethanol. Under the conditions of the present study, MOE supplementation in lactating ewes had no effect on milk yield and milk composition. One explanation for the lack of MOE effects on milk production traits may be the number of animals used in the study or the mild concentrations of treatments. In either case, the objective of this study was accomplished, as the different MOE doses were intended not to have negative effects on milk production traits.

### 4.3. Ewe Performance Traits

In this study, no differences in animal performance were observed between treatments. However, some authors reported that supplementation with leaf extracts of different plants with powerful antioxidant activity in moderate doses had positive effects on ADG [3], milk yield, oxidative stability in products such as milk and meat and attenuation of diseases related to oxidative stress, such as mastitis or heat stress [13,40]. Under the conditions of this study, MOE did not cause significant changes in ewe and lamb preweaning performance. However, it is important to note that some other variables, such as apparent nutrient digestibility, ruminal fermentation parameters and blood metabolic profiles, may have been affected but were not analyzed. Further research efforts should analyze the effects of MOE on rumen function and blood metabolites.

Several authors reported decreases in DMI when lactating sheep were supplemented with high amounts of polyphenols (especially condensed tannins). However, goats were reported to possess greater ability to tolerate and digest these compounds compared to sheep [4]. Likewise, Kholif et al. [1] supplemented lactating Nubian goats with 10, 20 and 40 mL/day of MOE obtained from fresh leaves in aqueous solution and reported a linear improvement of DMI, however, the supplementation had no effect on the performance of goats (IBW, FBW or BWC). It should be noted that Kholif et al. [1] used aqueous MOE obtained from fresh leaves and that the extract was stored at 4 °C, whereas in the present study, MO leaves were dried at 40 °C for 72 h and the hydroalcoholic extract was stored at 20 °C. Also, a study carried out by Adegun and Aye [41] in West African Dwarf Rams sheep reported that supplementation of *Pannicum maximum* with MO meal had no effect on the IBW, FBW and BWC, but DMI decreased as MO supplementation levels increased. On the other hand, Kekana et al. [14] microsupplemented Jersey cows with 0, 30 and 60 g of MO meal per animal per day and did not observe differences in DMI and BWC. In the present study, the animals’ basal diets allowed them to meet their nutritional requirements, which was reflected in a positive performance throughout the experiment. In addition, the ewes gained (*p* < 0.05) BW and BCS, indicating that over the experiment they were in a positive energy balance.

In this study, even though overall performance and milk production traits were not changed, some changes may have occurred in the rumen microflora, as reported by Dong et al. [2]. These authors supplemented lactating Holstein cows with different levels of MO foliage and reported that milk yield and energy-corrected milk were similar between treatments. They also reported an increase in the abundance of the *Methanosphaera* genus and *Methanosphaera* sp. ISO3-F5, which was induced by secondary metabolites of MO in the diet, suggesting that MO supplementation not only improves energy density in milk (fat content), but could also be used as a potential additive to reduce methane emissions.

### 4.4. Lamb Performance Traits

It was reported that preweaning is a very important stage in all production systems focused on meat production [26]. In this study, supplementation of MOE in lactating ewes did not negatively affect growth performance and weaning weight (LWW) in suckling lambs. This is very important for lamb performance, since ewe milk is the only food source during the early life of a lamb [26]. Preweaning growth essentially depends on ingested energy, but this was not evaluated in this study. However, if maternal milk was provided to lambs in low amounts of poor nutritional quality, the lamb growth rates and their survival would have deteriorated during the preweaning period [26].

No significant effects on milk energy intake in lambs (4.25 ± 1.53 MJ/d, data not presented) were observed. Although little is known regarding energy intake from sheep receiving MOE, one study reported that metabolizable energy intake in ewe’s increases with MO leaves supplementation [13]. The energy partition and utilization from sheep receiving plant parts or extracts from MO remains unknown.

The preweaning performance traits found in this study were higher (220 to 250 g/d) then those reported by Chay-Canul et al. [26], who fed animals in similar conditions and reported ADG of lambs from 150 to 160 g/d. On the other hand, Guzman et al. [42] and McGrath et al. [40] reported that antioxidant supplementation may help prevent negative effects of oxidative stress associated with growth in young ruminants, thereby reducing the incidence of morbidity and mortality and ultimately improving their performance. Also, Guzman et al. [42] supplemented lactating goats with concentrates rich in orange byproducts as a strategy to supply antioxidants and found no differences between the treatments in terms of BW and LWW in their suckling kids.

Another strategy to improve lamb performance is to increase energy density in dam diets. In this regard, Titi and Al-Fataftah [43] supplemented lactating Awassi ewes with 3% and 5% soybean or sunflower oil and evaluated the effects on performance and on their suckling lambs. They reported no improvements in initial or final ewe BW or changes in these levels, and milk intake of lambs decreased in comparison to the control diet (no fat supplemented). Average daily gains (286–314 g d^−1^) were not affected by dietary supplementation with oils and the milk conversion ratio (kg milk/kg ADG) only varied with sunflower oil at 5% DM. Gallardo et al. [44] evaluated the effect of supplementing linseed oil (3%) and natural or synthetic vitamin E in lactating Churra ewes with their lambs and observed that oil supplementation did not affect ADG, BW or LWW of suckling lambs. Under tropical and subtropical conditions, perhaps the best feeding strategy is to use extracts and byproducts from native plants [45]. In this context, even though the use of oils was reported to increase dietary energy density and improve lamb performance [44], the use of plant extracts may be an appropriate choice as they are available and easy to mix into diets.

When using plant extracts, it is important to note that animal responses may vary depending on their bioactive compound contents. In the present study, despite the high proportion of phenolic acids (140.41 ± 3.40) expressed in GAE 100 mL^−1^ of MOE and condensed and hydrolyzed tannins (7.77 ± 0.13, 182.55 ± 11.03), expressed as mg CE or TAE 100 mL^−1^ of MOE, no increases in milk yield or composition or performance of lambs or their mothers were observed.

Further studies should consider studying the effects of MOE on oxidative stress, blood parameters and quality of animal products. Also, higher doses of MOE and increased numbers of animals per treatment should be included.

## 5. Conclusions

Overall, the results from this study showed that dietary supplementation of hydroalcoholic extracts of *Moringa oleifera* leaves at doses of 40 or 60 mL/ewes/d in lactating ewes did not have negative effects on milk yield, milk composition or lamb performance. For greater scientific reach, further studies should consider the use of *Moringa oleifera* focusing on rumen function, blood metabolites and fatty acid profiles of animal products, such as meat, milk and dairy products.

## Figures and Tables

**Table 1:** Chemical composition (g/kg of dry matter) of the concentrate and forage offered to lactating ewes.

Parameter	Concentrate	Forage
Dry matter	900	283
Crude protein	150	31
Neutral detergent fibre	438	693
Acid detergent fiber	160	470
Organic matter	963	953
Ether extract	43	19.2
Metabolizable energy (MJ/kg DM) *	11.5	7.6

* Estimated from [22].

**Table 2:** Condensed tannins, hydrolyzable tannins, total phenolic compounds, total flavonoid contents and antioxidant activity (DPPH and ABTS) of *Moringa oleifera* leaf hydroalcoholic extract.

Compound	Amount (Mean ± SE)
Total saponins (mg DE/100 mL)	21.39 ± 1.40
Condensed tannins (mg CE/100 mL)	7.77 ± 0.13
Hydrolysable tannins (mg TAE/100 mL)	182.55 ± 11.03
Total phenols (mg GAE/100 mL)	140.41 ± 3.40
Total flavonoid (mg QE/100 mL)	3.72 ± 0.19
DPPH (mM trolox equivalent/100 mL)	32.63 ± 3.74
ABTS (mM trolox equivalent/100 mL)	200.13 ± 8.84

Data are represented as mean ± SE of three measurements. DE: Diosgenin equivalent; CE: cathechin equivalent; TAE: tannic acid equivalent; GAE: gallic acid equivalent; QE: quercetin equivalent.

**Table 3:** Milk yield and composition in Pelibuey × Katahdin ewes supplemented with *Moringa oleifera* leaf extract.

Variable	Daily *Moringa oleifera* Extract Supplementation (mL)				SE	*p*-Value
	0	20	40	60		
Total milk yield (kg)	48.7	49.2	57.1	53.4	5.10	0.66
Daily milk yield (kg)	1.15	1.13	1.28	1.23	0.12	0.87
Milk composition						
Fat (%)	6.27	6.11	6.09	6.01	0.08	0.13
Protein (%)	4.24	4.33	4.21	4.52	0.09	0.12
Daily yields						
Fat-corrected milk (kg/d)	0.79	0.85	0.89	0.95	0.08	0.59
Energy-corrected milk (kg/d)	0.74	0.77	0.83	0.87	0.07	0.68
Fat yield (kg/d)	0.04	0.04	0.05	0.05	0.004	0.43
Protein yield (kg/d)	0.05	0.05	0.05	0.05	0.004	0.79

Fat-corrected milk and energy-corrected milk were calculated according to [24,25]. SE: Standard error.

**Table 4:** Preweaning ewe and lamb performance traits in Pelibuey × Katahdin ewes supplemented with *Moringa oleifera* leaf extracts.

Variable	Daily *Moringa oleifera* Extract Supplementation (mL)				SE	*p*-Value
	0	20	40	60		
Dry matter intake (kg/d)	2.08 ^a,b^	1.98 ^a,b^	2.37 ^a^	1.89 ^b^	0.09	0.02
Dry matter intake (g/kg ^0.75^/d)	137 ^a,b^	125 ^b,c^	143 ^a^	121 ^c^	3.08	<0.001
Initial body weight (kg)	34.6	37.3	38.1	36.4	2.20	0.72
Final body weight (kg)	40.7	42.3	46.9	42.2	2.41	0.38
Body weight change (kg/d)	0.137	0.111	0.195	0.129	0.02	0.28
Litter weaning weight (kg)	14.2	13.7	15.1	13.7	0.57	0.38
Ewe efficiency (%)	35.2	32.7	32.2	33.1	1.64	0.63
Birth weight (kg)	3.66	3.52	3.82	3.30	0.25	0.55
Average daily preweaning gain in lambs (kg/d)	0.23	0.22	0.25	0.23	0.01	0.30

Body weight change: Change in ewe body weight per day during lactation calculated as (final body weight − initial body weight)/45 days. Ewe efficiency was calculated as (litter weaning weight/final body weight) × 100. Means in the same row with different superscripts (a, b, c) are different (*p* < 0.05). SE: Standard error.
